# Work Related Stress, Burnout, Job Satisfaction and General Health of Nurses

**DOI:** 10.3390/ijerph120100652

**Published:** 2015-01-12

**Authors:** Natasha Khamisa, Brian Oldenburg, Karl Peltzer, Dragan Ilic

**Affiliations:** 1 Department of Public Health, School of Health Sciences, Monash South Africa, 144 Peter Road, Roodepoort, 1725 Johannesburg, South Africa; 2 Department of Epidemiology and Preventive Medicine, Faculty of Medicine, Nursing and Health Sciences, Monash University, Victoria, 3004 Melbourne, Australia; E-Mail: dragan.ilic@monash.edu; 3 School of Population and Global Health, University of Melbourne, Victoria, 3010 Melbourne, Australia; E-Mail: brian.oldenburg@unimelb.edu.au; 4 Department of Psychology, University of Free State, Bloemfontein 9300, South Africa; 5 ASEAN Institute for Health Development, Mahidol University, Salaya, Phutthamonthon, Nakhonpathom 73170, Thailand; 6 HIV/AIDS/STIS and TB Research Unit (HAST), Human Sciences Research Council, Pretoria 0001, South Africa; E-Mail: kpeltzer@hsrc.ac.za

**Keywords:** burnout, general health, job satisfaction, nurses, work related stress

## Abstract

Gaps in research focusing on work related stress, burnout, job satisfaction and general health of nurses is evident within developing contexts like South Africa. This study identified the relationship between work related stress, burnout, job satisfaction and general health of nurses. A total of 1200 nurses from four hospitals were invited to participate in this cross-sectional study (75% response rate). Participants completed five questionnaires and multiple linear regression analysis was used to determine significant relationships between variables. Staff issues are best associated with burnout as well as job satisfaction. Burnout explained the highest amount of variance in mental health of nurses. These are known to compromise productivity and performance, as well as affect the quality of patient care. Issues, such as security risks in the workplace, affect job satisfaction and health of nurses. Although this is more salient to developing contexts it is important in developing strategies and intervention programs towards improving nurse and patient related outcomes.

## 1. Introduction

Described as a state of physical and emotional depletion, burnout is a result of prolonged exposure to stressful working environments [1]. As such, burnout among nurses in particular has been reported to be higher than other health professionals owing to the nature of their work [2]. Nursing requires the delivery of humane, empathetic, culturally sensitive, proficient and moral care, in working environments with limited resources and increasing responsibilities. Such imbalance between providing high quality care and coping with stressful working environments can lead to burnout [3].

Work related stress is associated with burnout, job satisfaction and physical as well as mental health outcomes [3,4,5]. Stressors contributing to the experience of work related stress, including poor supervision, conflict with peers and patients, high job demands [6,7] and overtime [8] are all associated with one or more dimensions of burnout. The Maslach Burnout Model [9,10] postulates that prolonged exposure to environmental and situational stressors resulting in work related stress, contributes to emotional exhaustion, depersonalization and a lack of personal accomplishment. Similarly, work related stress resulting from stressors, such as higher workloads, as well as staff issues, including lack of resources, has been found to be associated with poor job satisfaction [11,12]. Spector attributes this to a mismatch between job expectations and actual working environments, which contributes to higher levels of work related stress and lower levels of job satisfaction [13]. Research confirms higher levels of job satisfaction within less stressful working environments [5]. Stressors such as poor patient outcomes, conflict with peers, high workload and job demands as well as poor supervision and lack of support are all associated with poor physical and mental health outcomes [14,15,16]. Cannon’s stress theory [17,18] explains this response as an imbalance in homeostasis, whereby prolonged exposure to stressors results in a breakdown of the biological system. This breakdown prevents compensatory and anticipatory changes that aid in coping, thereby resulting in poor health outcomes such as headaches, insomnia, social dysfunction and depression [4,19,20].

Important to note is that burnout and job satisfaction have also been found to be associated with each other as well as poor health outcomes. Although not studied extensively in relation to work related stress, existing research shows a negative relationship between burnout and job satisfaction [3]. According to the conservation of resources theory [21,22], burnout directly affects health outcomes through the depletion of resources necessary for coping, thereby leading to negative states of being characterized by exhaustion, fatigue, somatization and social withdrawal [23].

With grave impact on work productivity, patient care, staff attrition and turnover rates [24], a better understanding of existing relationships between work related stress, burnout, job satisfaction and general health of nurses is required. This is especially important in developing contexts, with majority of research exploring these relationships being conducted in developed contexts [25,26,27]. Moreover, as a consequence of uniquely stressful work environments, burnout among South African nurses is reported to be higher than nurses working in other countries [24,28,29]. Researchers have compared the degree of emotional exhaustion, depersonalization and personal accomplishment among South African nurses with nurses from eight different countries (USA, Canada, UK, Germany, New Zealand, Japan, Russia and Armenia) [30,31]. Average burnout scores on each subscale of the Maslach Burnout Inventory showed a higher degree of emotional exhaustion (27.0) and depersonalization (11.1) with lower levels of personal accomplishment (21.9) among South African nurses [30]. This study aimed at identifying relationships between work related stress, burnout, job satisfaction and general health among nurses.

## 2. Experimental Section

### 2.1. Participants

Of a total of 1200 nurses who were invited, 895 nurses participated (response rate = 75%). Overall, majority of participants were black (46%) females (85%), from private hospitals (59%). A total of 28% were above the age of 50, 72% had a diploma/certificate and 27% had more than 25 years work experience. The majority of the participants worked between 4 to 6 days and 31 to 40 h per week. The demographic data obtained suggests that nurses working in private hospitals were 51 years and older (51%), white (84%), females (68%), with postgraduate degrees (62%) and more years of experience (62%).

### 2.2. Procedure

This study was conducted in the year 2013. All subjects gave their informed consent for inclusion before they participated in the study. The study was conducted in accordance with the Declaration of Helsinki, and the protocol was approved by the Ethics Committee of Human Sciences Research Council (HSRC) (REC 3/20/03/12). Following ethics approval from the Human Sciences Research Council (HSRC) as well as permission from the Gauteng Department of Health (South Africa), four hospitals across the province were selected using stratified random sampling. All private and public hospitals in Gauteng province were stratified by area and size after which two private and two public hospitals were randomly chosen. Permission to conduct the study was obtained from hospital and nurse managers at each of the chosen hospitals.

For this study, unit managers were requested to distribute invitation packs containing an explanatory statement, consent form, five questionnaires and a sealable envelope. Invitation packs were distributed randomly by unit managers to a total of 300 nurses per hospital.

Participants were given 3 weeks to complete the questionnaires. Reminders were issued verbally and in the form of posters placed on notice boards two weeks after initial questionnaire distribution. Participants were requested to return the completed questionnaires together with signed informed consent forms in sealable envelopes provided to them. The sealed envelopes were to be placed in sealed boxes situated around the hospital. These were only accessible to the researcher.

### 2.3. Measurement Tools

All participants received five questionnaires consisting of a socio-demographic questionnaire (SDQ), Nursing Stress Inventory (NSI), Maslach Burnout Inventory—Human Services Survey (MBI-HSS), Job Satisfaction Survey (JSS) and General Health Questionnaire (GHQ-28) [9,13,32,33,34].

The SDQ consisted of questions pertaining to the age, gender, level of education, level of experience, population group, and number of days/hours worked per week.

The NSI was designed to specifically measure the frequency and severity of five specific stressors identified among South African nurses (patient care, staff issues, supervision/management issues, overtime and job demands). Containing 78 items, the first 39 statements were rated in terms of perceived intensity of the particular stressor on a 9-point scale, ranging from 1 (*low*) to 9 (*high*). In the second part of the questionnaire, participants rated the remaining 39 statements on a 10-point scale ranging from 0 (*no days*) to 9+ (*more than 9 days*) in terms of the frequency with which they experienced the stressors over the previous 6 months. Validity and reliability of the NSI was tested among 1780 professional, enrolled and auxiliary nurses from seven of the nine provinces in South Africa. Findings confirmed the NSI as a reliable and valid measure of stress among South African nurses with Cronbach alpha coefficients ranging between 0.91 and 0.93 [35]. The mean inter-item correlation coefficients were in the recommended range (0.15 < *r* < 0.50) [32].

The MBI-HSS was designed to measure burnout among individuals working in the human services and health care occupations including nursing. Consisting of 22 items in the form of statements based on personal feelings and attitudes, this questionnaire contains three subscales. The emotional exhaustion (EE) subscale (9 items) includes statements such as “I feel emotionally drained from my work”, the depersonalization (DP) subscale (5 items) includes statements such as “I feel I treat some recipients as if they were impersonal objects” and the personal accomplishment (PA) subscale includes statements such as “I have accomplished many worthwhile things in this job”. This tool is a reliable measure of burnout among nurses with Cronbach alpha values of 0.90 for EE, 0.71 for DP and 0.79 for PA [9]. It has also been found to be reliable among South African nurses with Cronbach alpha coefficients exceeding 0.70 for all subscales (emotional exhaustion 0.78, depersonalization 0.74 and personal accomplishment 0.75) [29].

The JSS measures job satisfaction within organizations in the social sector of employment. Consisting of 36 items the questionnaire has nine facets (pay, promotion, supervision, fringe benefits, contingent rewards, operating conditions, coworkers, nature of work and communication). Responses are indicated in terms of how true the statement is (higher scores indicate “truer” statements) based on a six point rating scale ranging from “disagree very much” (1) to “agree very much” (6). The JSS is a reliable instrument with Cronbach alpha coefficients exceeding 0.70 for all the facets [13,33]. The JSS has been found to be reliable among nurses (Cronbach alpha = 0.89) and shows good construct validity [36]. This tool has also been found to possess acceptable reliability across cultures (Cronbach alpha > 0.90 in 17 countries including South Africa) [37].

The GHQ-28 was developed to detect psychiatric illness as well as current psychological states and perceived quality of life. Undesirable psychological states can have consequences for physical health. This instrument consists of 28 items measured on four sub-scales (somatic symptoms (SS), anxiety and insomnia (AS), social dysfunction (SD) and severe depressive symptoms (DS)). The SS subscale includes items, such as “been feeling run down” and “been getting pains in the head”. The AS subscale contains items, such as “lost much sleep over worry” and “feeling nervous and strung up all the time”. The SD subscale contains items, such as “taking longer on things” and “enjoying normal day to day activities”. The DS subscale includes items, such as “feeling worthless” and “feeling that life is hopeless”. Responses for positive items are scored from 0 (always) to 3 (never) and negative items are scored from 3 (always) to 0 (never). A high score indicates poor general health and vice versa. This tool is reliable with Cronbach alpha values ranging between 0.69 and 0.90 for all sub-scales [34]. The GHQ has been found to be reliable (Cronbach alpha between 0.70 and 0.83 for all sub-scales) and valid (*p_close fit_* = 1.00) among South African samples [38] including nurses (Cronbach alpha = 0.84) [39].

### 2.4. Design and Analysis

This study cross sectionally measured work related stress, burnout, job satisfaction and general health of nurses. Data analysis using IBM-SPSS Statistics Version 20 included descriptive statistical analysis to determine frequencies. Multiple linear regression analysis was used to determine significant associations between variables. The stressors contributing to work related stress were entered into models with facets of burnout, job satisfaction and general health. Burnout was also entered as an independent variable into models with job satisfaction and general health. Job satisfaction was entered as an independent variable into a model with general health as the dependent variable. Missing data were handled using listwise deletion. Assumptions of normality and linearity were confirmed using histograms and partial plots. Independence of errors was confirmed using the Durbin-Watson test (values between 1 and 3). Homoscedasticity was confirmed using *ZRESID (Y-axis) and *ZPRED (X-axis) plots.

## 3. Results and Discussion

### 3.1. Results

#### 3.1.1 Work Related Stress and Burnout

Multiple linear regression revealed that staff issues contributing to work related stress is best associated with each dimension of burnout. All models were significantly different from zero (*p* < 0.05). Staff issues explain the highest variance in emotional exhaustion (16%), depersonalization (13%) and personal accomplishment (10%) as shown in Table 1. This was after controlling for age, gender, population group, education, experience and hospital type (public and private), which explained an additional variance of 12% in emotional exhaustion, 10% in depersonalization and 1% in personal accomplishment.

#### 3.1.2. Work Related Stress and Job Satisfaction

Associations were found between satisfaction with pay and patient care as well as overtime, each explaining 12% variance. Satisfaction with promotion is best associated with patient care explaining 9% variance. Satisfaction with supervision is best associated with supervision/management issues explaining 15% variance. Satisfaction with fringe benefits is best associated with patient care explaining 10% variance. Satisfaction with contingent rewards is best associated with staff issues explaining 12% variance. Satisfaction with operating conditions is best associated with patient care explaining 12% variance. Satisfaction with coworkers is best associated with staff issues explaining 14% variance. Satisfaction with the nature of work is best associated with patient care and staff issues, each explaining 3% variance. Satisfaction with communication is best associated with staff issues explaining 17% variance. All associations are significant (*p* < 0.05). Of all the factors, the highest amount of variance in job satisfaction is explained by stress related to staff issues after controlling for age, gender, population group, education, experience and hospital type (public and private), which explained an additional variance of 9% in satisfaction with communication. This is shown in Table 2.

**Table 1:** Staff issues and burnout.

Variables	B	SE B	β	*t*-Statistic
Staff Issues and Emotional Exhaustion				
Staff Management	0.29	0.04	0.30	7.42 *
Inadequate and Poor Equipment	0.12	0.04	0.12	3.04 *
*R*^2^ = 0.16, *F* (9, 856) = 17.70, *p* < 0.05. * *p* < 0.05				
Staff Issues and Depersonalization				
Staff Management	0.17	0.03	0.20	4.90 *
Stock Control	0.09	0.03	0.11	2.83 *
Poorly Motivated Coworkers	0.09	0.04	0.10	2.31 *
*R*^2^ = 0.13, *F* (9, 856) = 13.55, *p* < 0.05. * *p* < 0.05				
Staff Issues and Personal Accomplishment				
Adhering to Hospital Budget	−0.19	0.03	−0.22	−5.44 *
Staff Management	−0.14	0.04	−0.16	−3.80 *
Meeting Deadlines	−0.07	0.04	−0.08	−1.97 *
*R*^2^ = 0.10, *F* (9, 856) = 10.73, *p* < 0.05. * *p* < 0.05				

**Table 2:** Staff issues and satisfaction with communication.

Variables	B	SE B	β	*t*-Statistic
Stock Control	−0.19	0.03	−0.23	−6.23 *
Inadequate and Poor Equipment	−0.21	0.03	−0.23	−6.14 *
Adhering to Hospital Budget	−0.13	0.03	−0.16	−4.06 *
Staff Management	−0.10	0.04	−0.12	−2.85 *
Security Risk in Area of Job	−0.08	0.03	−0.09	−2.41 *
*R*^2^ = 0.17, *F* (9, 856) = 19.90, *p* < 0.05. * *p* < 0.05				

#### 3.1.3. Work Related Stress and General Health

Multiple linear regression revealed that staff issues are best associated with somatic symptoms, social dysfunction and severe depressive symptoms, explaining 7%, 11% and 5% variance, respectively. Patient care is best associated with anxiety and insomnia explaining 11% variance. All associations are significant (*p* < 0.05). Both patient care and staff issues are best associated with general health after controlling for age, gender, population group, education, experience and hospital type (public and private), which explained an additional variance of 8% in anxiety/insomnia as well as social dysfunction. This is shown in Table 3.

**Table 3:** Patient care with anxiety/insomnia and staff issues with social dysfunction.

Variables	B	SE B	β	*t*-Statistic
Patient Care and Anxiety/Insomnia				
Inadequate Information About Patient From Doctor	0.08	0.02	0.16	3.87 *
Disagreement With Doctor About Treatment of Patient	0.07	0.02	0.14	3.00 *
Making A Mistake When Treating Patient	0.06	0.02	0.12	2.70 *
Death Of Patient	0.06	0.02	0.13	2.63 *
*R*^2^ = 0.11, *F* (8, 853) = 13.11, *p* < 0.05. * *p* < 0.05				
Staff Issues and Social Dysfunction				
Staff Management	0.10	0.02	0.29	6.97 *
Inadequate And Poor Equipment	0.05	0.01	0.14	3.42 *
Security Risk In Area of Job	0.04	0.01	0.12	3.15 *
Poorly Motivated Coworkers	0.05	0.02	0.14	3.12 *
Adhering To Hospital Budget	0.04	0.01	0.12	2.84 *
Meeting Deadlines	0.03	0.01	0.09	2.18 *
Stock Control	0.03	0.01	0.08	2.05 *
*R*^2^ = 0.11, *F* (9, 856) = 11.77, *p* < 0.05. * *p* < 0.05				

#### 3.1.4. Burnout and Job Satisfaction

Multiple linear regression revealed that emotional exhaustion and satisfaction with pay are associated explaining 7% variance. All three subscales are associated with satisfaction with promotion explaining 4% variance. Emotional exhaustion as well as personal accomplishment and satisfaction with supervision are associated explaining 2% variance. Associations between all three subscales and satisfaction with fringe benefits were found explaining 9% variance. Emotional exhaustion and personal accomplishment are associated with satisfaction with contingent rewards explaining 9% variance. All three subscales are associated with satisfaction with operating conditions, coworkers and nature of work explaining 7%, 10% and 10% variance, respectively. Emotional exhaustion and satisfaction with communication are associated explaining 14% variance. All associations are significant (*p* < 0.05). Emotional exhaustion is best associated with job satisfaction with communication after controlling for age, gender, population group, education, experience and hospital type (public and private), which explained an additional variance of 5% in satisfaction with communication. This is shown in Table 4.

**Table 4:** Emotional exhaustion and satisfaction with communication.

Variable	B	SE B	β	*t*-Statistic
Emotional Exhaustion	−0.32	0.04	−0.36	−8.73 *
*R*^2^ = 0.14, *F* (3, 850) = 44.38, *p* < 0.05. * *p* < 0.05				

#### 3.1.5. Burnout and General Health

Multiple linear regression revealed that emotional exhaustion and personal accomplishment are associated with somatic symptoms explaining 21% variance. Emotional exhaustion and depersonalization are associated with anxiety/insomnia as well as social dysfunction explaining 31% and 14% variance, respectively. Emotional exhaustion is associated with severe depressive symptoms explaining 4% variance. All associations are significant (*p* < 0.05). Emotional exhaustion and depersonalization explain the highest amount of variance in anxiety/insomnia after controlling for age, gender, population group, education, experience and hospital type (public and private), which explained an additional variance of 4% in anxiety/insomnia. This is shown in Table 5.

**Table 5:** Burnout and Anxiety/Insomnia.

Variables	B	SE B	β	*t*-Statistic
Emotional Exhaustion	0.24	0.02	0.50	13.67 *
Depersonalization	0.04	0.02	0.08	2.06 *
*R*^2^ = 0.31, *F* (3, 850) = 129.33, *p* < 0.05. * *p* < 0.05				

#### 3.1.6. Job Satisfaction and General Health

Multiple linear regression revealed that satisfaction with promotion, supervision, fringe benefits, coworkers, nature of work and communications are all associated with somatic symptoms explaining 15% variance. Anxiety/insomnia is associated with promotion, contingent rewards, operating conditions, coworkers, nature of work and communication, explaining 17% variance. Social dysfunction is associated with supervision, fringe benefits, contingent rewards, operating conditions, nature of work and communication explaining 15% variance. Depression is associated with nature of work explaining 4% variance. All associations are significant (*p* < 0.05). Promotion, contingent rewards, operating conditions, coworkers, nature of work and communication explain the highest amount of variance in anxiety/insomnia after controlling for age, gender, population group, education, experience and hospital type (public and private) which explained an additional variance of 9% in anxiety/insomnia. This is shown in Table 6.

**Table 6:** Job Satisfaction and Anxiety/Insomnia.

Variables	B	SE B	β	*t*-Statistic
Coworkers	−0.11	0.02	−0.21	−6.01 *
Communication	−0.09	0.02	−0.16	−4.49 *
Nature of Work	−0.08	0.03	−0.11	−3.29 *
Promotion	−0.05	0.02	−0.09	−2.24 *
Contingent Rewards	−0.04	0.02	−0.09	−2.31 *
Operating Conditions	−0.04	0.02	−0.08	−2.30 *
*R*^2^ = 0.17, *F* (9, 841) = 18.67, *p* < 0.05. * *p* < 0.05				

### 3.2. Discussion

This study examined the relationships between work related stress, burnout, job satisfaction and general health of nurses. Of the five stressors contributing to work related stress, staff issues was found to be most associated with burnout as well as job satisfaction. Burnout explained the highest amount of variance in general health of nurses.

Existing literature confirms that staff issues including excessive administration, stock control and colleagues not doing their job influences the levels of stress experienced by nurses [32,40]. Staff issues have been reported as one of the most significant stressors among nurses [28]. In one such study, professional, enrolled and auxiliary nurses reported severe stress due to staff issues [32]. This can be explained by the overburdened South African health system where nurses may be unable to meet the demands of their job due to poor staff management which may negatively affect morale [41], lack of resources which may negatively affect patient care [29] and security issues owing to high levels of crime in the country [42].

This study found that stress related to staff issues in particular was associated with all three dimensions of burnout explaining 16% variance in emotional exhaustion, 13% variance in depersonalization and 10% variance in personal accomplishment. In support of these findings, studies conducted in developed contexts have found staff issues such as poor staff management and resource inadequacy to be associated with emotional exhaustion, depersonalization and personal accomplishment [43]. In this study, poor staff management as well as poor or inadequate equipment explained the highest amount of variance in burnout (16%). These issues have been reported in several news articles, including the New York Times [44] and News24 [45], however, empirical evidence for this association has been shortcoming, with minimal impact on nursing policies and practices.

Stress related to staff issues and job satisfaction with communication were also found to be associated. Although research has shown that staff issues such as poor staff management and resource inadequacy are associated with job satisfaction [43], the findings of this study revealed that security risks in the workplace also play a role in influencing job satisfaction among nurses. Within the South African context, political violence is surpassed by high levels of violent crime. As such feelings of insecurity and fear become predominant. This can be understood from Maslow’s needs hierarchy, whereby in order to satisfy higher level needs, lower level needs such as safety must first be satisfied. In the event that an individual’s safety and security is in danger, patient care becomes less important [46].

Security risks in the workplace, has also been found to affect general health of nurses [47,48]. The findings of this study show that security risks in the workplace as well as other staff issues including poor staff management and resource inadequacy explains 11% variance in social dysfunction. This means that nurses experiencing work related stress as a result of staff issues are more likely to feel incapable of enjoying activities and engaging in healthy social behavior. This prevents adaptational outcomes, such as psychological wellbeing and good somatic health [49]. Furthermore, stress related to patient care is equally important in influencing mental health among nurses (explaining 11% variance). This is supported by research showing that poor communication with doctors, negative patient outcomes and mistakes when treating patients are all associated with mental health problems [14]. Burnout (emotional exhaustion) and job satisfaction (with communication) demonstrate a negative relationship in this study. Limited research shows that opportunities for communication about stress provoking issues provides a buffer for emotional exhaustion, which results when coping resources become depleted [3].

Based on the findings of this study, stress related to staff issues is associated with burnout, job satisfaction and general health of nurses. The major staff issues identified in this study include poor staff management, resource inadequacy and security risks in the work place. According to Fretwell, staff management is important given that the managers’ decisions influence the working environment [50]. Poor staff management therefore diminishes the staff morale, which leads to feelings of depersonalization (burnout) as well as job dissatisfaction [51]. Moreover, lack of resources invokes feelings of insecurity about obtaining and maintaining resources necessary for meeting job demands, thereby triggering stress, which manifests in burnout [40]. Lack of essential resources, such as treatment equipment, medication and examination facilities, compromises patient care and negatively affects job satisfaction [5]. In the context of South Africa, security risks in the workplace involve situations of violence and crime, whereby nurses are exposed to dangerous situations on their way to work and while at work [52]. This prompts stressful responses, which eventually leads to burnout. As such nurses are restricted from working to their full potential, thereby negatively affecting their levels of job satisfaction [5] and their health [53].

In addition to stress related to staff issues, job satisfaction is also significantly associated with general health. However, both variables explain a smaller amount of variance in general health compared to burnout in this study. Although the association between burnout and poor physical, mental as well as social health outcomes has been confirmed in many studies [54,55,56,57,58], this study shows that burnout is most strongly associated with anxiety/insomnia. With mental health problems reported among the top ten work related consequences, these findings can be understood as the depletion of mental energy (emotional exhaustion) and mental distancing (depersonalization), thereby compromising nurses’ ability to perform tasks and resulting in anxiety/insomnia [48].

Stress prevention strategies are both person level as well as organization level. Stress management programs involving education and training on coping with stress is a person level strategy that provides support for nurses dealing with stress related outcomes. Organization centered approaches address work related stressors by reducing or eliminating them through better management of nurses and provision of adequate resources [59]. These have been found to be successful in work settings, whereby stress in the workplace is not viewed as a weakness but rather a phenomenon that can be managed by creating a culture of openness and understanding [53]. It is recommended that an integrated stress prevention strategy of both person centered and organizational centered approaches be used to address work related stress, burnout, job dissatisfaction and poor general health among nurses. This should involve input from nurses as well as management in order to ensure collective commitment towards improving nurse and patient related outcomes.

These findings provide some empirical evidence confirming the relationship between work related stress, burnout, job satisfaction and general health of nurses in a developing country context. Although issues related to security risks in the workplace may be more salient within the context of a developing country like South Africa, these study findings also confirm a number of the findings that have been previously identified from studies undertaken in more developed countries. Poor staff management and resource inadequacy are associated with burnout, job satisfaction and general health of nurses in both developing and developed contexts. It is very important to identify and delineate very clearly which factors are particularly salient and relevant to developing countries because it is very important to develop strategies and intervention programs, which can either prevent or at least ameliorate these. It is also equally important to recognize that the role of work related stress, burnout, job satisfaction and general health of nurses on poor patient outcomes, high turnover, low retention, poor job performance, absenteeism and increased healthcare costs are also evident within an international context.

Several studies have shown that intervention strategies such as additional training in identification and management of work related stress through assertiveness and relaxation have successfully improved job performance levels among nurses. Support groups as well as process consultation with nurse managers have also been effective in solving problems across interdisciplinary staff teams [60]. The effect of such interventions on staff absenteeism and turnover is not well researched and should be explored in the context of South Africa.

Limitations include generalizability (based on one province) and study design (cross sectional). However, it is the intention of the authors to use this study as a foundation for further evaluation of the same sample at a later stage to determine causality. Given that this study included a sample of nurses from one province in South Africa, it would be useful to replicate this study among South African nurses across the other provinces to compare findings and improve generalizability. Future research should also examine reverse relationships between the variables. Personal stressors including family problems, financial status and difficult relationships should also be studied in relation to work related stress, burnout, job satisfaction and general health of nurses. Differences between nurses working in public *vs.* private hospitals should be explored in future studies.

## 4. Conclusions

In conclusion, stress related to staff issues (including poor staff management, resource inadequacy and security risks) is most important in determining burnout and job satisfaction among nurses and possibly other health professionals. Burnout clearly impacts on the mental health and wellbeing of nurses, which is most likely compromising productivity, performance and the quality of patient care. Further research exploring specific strategies for managing stress and improving job satisfaction may reduce the impact of burnout on general health of nurses, while also minimizing absenteeism and turnover. This could be achieved through evidence based policies aimed at creating better work environments where nurses feel more secure and have adequate resources to successfully perform their jobs, hence improving their health outcomes as well as that of their patients.
